# Role of *XmnI*^*G*^ Polymorphism in Hydroxyurea Treatment and Fetal Hemoglobin Level at Isfahanian Intermediate β-Thalassemia Patients

**DOI:** 10.7508/ibj.2015.03.008

**Published:** 2015-07

**Authors:** Majid Motovali-Bashi, Tayyebeh Ghasemi

**Affiliations:** *Genetic Division, Dept. of Biology, Faculty of Sciences, University of Isfahan, Isfahan, Iran*

**Keywords:** Fetal hemoglobin (HbF), Hydroxyurea, Intermediate β-thalassemia

## Abstract

**Background:**

β-thalassemia is the most common monogenic disorder in human. The (CT) polymorphism at -158 upstream region of the *γ*^G^-*globin* gene and pharmacological factors such as hydroxyurea have been reported to influence *γ-globin* gene expression and the severity of clinical symptoms of β-thalassemia.

**Methods:**

In the present study, 51 β-thalassemia intermediate patients were studied*. Xmn1γ*^G^ polymorphism genotype was determined using Tetra-Primer ARMS-PCR technique. Hemoglobin (Hb) and fetal hemoglobin (HbF) levels were determined by gel electrophoresis.

**Results:**

Of 51 patients, 35 (68.6%) patients were heterozygous (CT) and 16 (31.4%) patients were homozygous (CC). Of 30 patients under treatment by hydroxyurea, 20 (66.7%) patients were heterozygous (CT) and 10 (33.3%) patients were homozygous (CC). Our results demonstrated that in the heterozygous (CT) genotype, the Hb (9.58 ± 1.25 gm/dl) and HbF (89.30 ± 21.87) levels were significantly higher in comparison with homozygous (CC) genotype (7.94 ± 1.34 gm/dl and 70.32 ± 40.56, respectively). Furthermore, we observed that after drug usage, the Hb and HbF levels in patients with heterozygous (CT) genotype (0.7 ± 1.26 gm/dl and 5.95±14.8, respectively) raised more in comparison with homozygous (CC) genotype (0.26 ± 1.43 gm/dl and 0.8 ± 1.31, respectively).

**Conclusion:**

Hb and HbF levels in the patients carrying T allele are increased significantly, and they also response to hydroxyurea treatment.

## INTRODUCTION

The β-thalassemia, which is the most common human single-gene disorder, is caused by any of more than 200 mutations in the β-globin gene [1]. These mutations lead to imbalanced synthesis of α- and β-globin chains, resulting in precipitation of excess peptide chains and hemolysis [2]. β-thalassemia is classified into three types: 1) β-thalassemia minor (trait) that often goes undiagnosed because people with the condition have no real symptoms other than mild anemia; 2) β-thalassemia major that patients have severe anemia (Cooley's anemia) and need blood transfusions throughout their life; 3) β-thalassemia intermediate that patients have varying effects from the disease [3].

Some genetic factors can influence the disease severity of β-thalassemia as follow: 1) The coinherit-ance of homozygosity or compound heterozygosity for mild β-thalassemia alleles; 2) coinheritance of α-thalassemia, which reduces the degree of α/β imbalance; 3) The genetic variation of patients that causes upregulation of *γ-globin* gene expression in adult life and therefore compensates the decreased levels of β-globin chain. The common genetic variant is C>T *XmnI* polymorphism (rs7482144) at the -158 site upstream region of the *γ*^G^*-globin* gene [4].

Temporal and tissue-specific transcription of the *β-globin* gene is regulated by the locus control region (LCR) consisting of five DNase I hypersensitive sites, which is located on 5′ site of the β*-globin* gene. These sites are devoid of nucleosome formation and therefore are more accessible to interactions with transcription factors. Transcriptional activators are bound to the LCR hypersensitive sites and then recall RNA polymerase II to these sites. Finally, RNA polymerase is loaded onto the gene to be transcribed [5]. The hypersensitive sites are needed to form an active chromatin loop with looping the β-globin locus.

This three-dimensional structure, which is termed an active chromatin hub, includes LCR elements interacting with transcriptional factors, downstream globin structural genes, and chromatin remodeling complexes necessary for hemoglobin (Hb) switching. These interactions in human adult and fetal cells activate the *β-globin* genes [6]. 

The -158 C > T polymorphism is located near a nuclease hypersensitive site at 50 to 150 bp upstream region of the *γ*^G^ gene. Perhaps the -158 substitution reduces the binding of transcription factor(s) that silence(s) the *γ-globin* gene expression in adult cells. Therefor, the *γ-globin* gene is reactivated in adult lifen [2, 7]. Furthermore, several pharmacologic agents, such as 5-azacytidine, erythropoietin, butyrates, and hydroxyurea have been shown to stimulate *γ-globin* gene expression *in vivo* and therefore might reduce the severity of clinical symptoms in patients with intermediate thalassemia [8]. Moreover, one study on β-thalassemia patients treated with hydroxyurea has revealed a significant correlation between the presence of T allele in *Xmn1* polymorphic site and the better treatment response. However, the molecular mechanisms responsible for this correlation have not been elucidated yet.

The main goal of this study is to investigate the association between *Xmn1γ*^G^ polymorphism and the amount of total Hb and fetal hemoglobin (HbF) in adults with intermediate β-thalassemia disease in Isfahan population as well as to investigate the association between *Xmn1γ*^G^ polymorphism and the effect of hydroxyurea. 

## MATERIALS AND METHODS

***Patients and DNA extraction. ***In the present study, 51 β-thalassemia intermediate patients were identified and studied. Mean age of the patients was 23 years. Cases were collected from Omid Hospital (Isfahan, Iran) between October 2011 to November 2012. The Hb and HbF levels were determined by electrophoresis (according to patients’ files). Patients' blood samples were taken according to the protocol approved by the Ethical Committee of Isfahan University of Medical Sciences (Isfahan, Iran). Venous blood samples were collected in EDTA-containing tubes and stored at -20°C. Genomic DNA was extracted using Miller's salting-out method [9] with slight modifications.

**Fig. 1:**
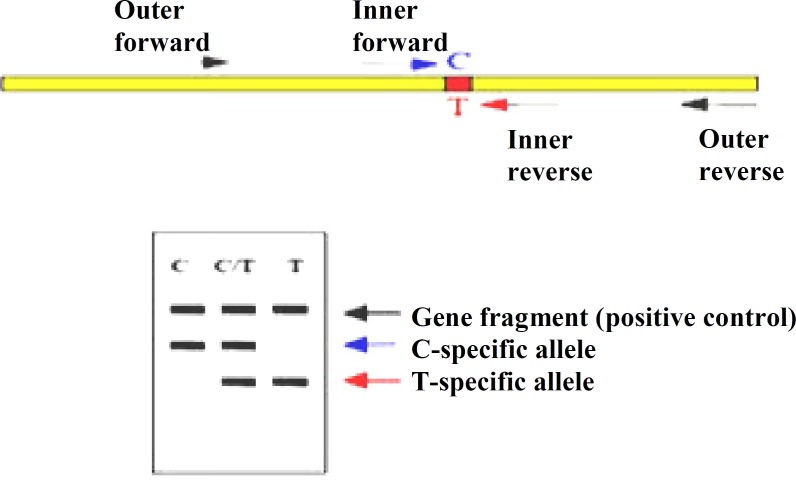
Diagrammatic representation of Tetra-Primer ARMS-PCR procedure

***Xmn1γ*** ^G^ ***polymorphism genotyping. ***In the current study, for *γ*^G^*-globin *(gi|28380636|ref|NG_000007.3) -158C/T polymorphism genotyping, two pairs of primers were designed for Tetra-Primer ARMS-PCR technique using SGD and NCBI databases and OLIGO 7 software . The reverse primers were designed in such a way that they paired particularly to different alleles of single nucleotide polymorphism. The outer primers could produce a large size product as a positive control in allele PCR reactions (Fig. 1). Primer sequences are shown in Table 1. PCR reaction was performed in a 25-μl volume, containing 0.024 nM DNA template (genomic DNA), 1.4 μl of 10× PCR buffer, 6 mM MgCl_2_, 0.6 mM mixed dNTP, 0.1 U/l taq DNA polymerase (Kawsar Biotech, Iran), 0.12 pmol/l each of outer forward or reverse primers, 0.28 pmol/l inner forward primer, and 0.6 pmol/l inner reverse primer (Kohangen Kowsar, Iran). The PCR cycling conditions were an initial denaturation at 95ºC for 3 min, followed by 30 cycles, at 95°C for 30 s, at 61°C for 60 s, at 72°C for 60 s, followed by 72ºC for 10 min. The PCR products were separated by electrophoresis on a 1.5% agarose gel. As expected, the C alleles were represented by DNA bands of 122 bp, the T alleles by a DNA band of 239 bp, and the control band by a DNA band of 317 bp. 

***Statistical analysis. ***Statistical analyses were performed using the SPSS 20 software package. The Chi-square test was used to evaluate case differences in the distribution of allele types and genotypes. The paired sample *t*-test was used to determine whether there was a significant difference between the average Hb and HbF levels before and after treatment with hydroxyurea. The independent *t*-test was also used to compare the average Hb and HbF levels between genotypes. In all cases, *P* < 0.05 was considered statistically significant.

**Table 1:** Tetra-Primer ARMS-PCR technique of the Xmn1 polymorphism

**PCR primers**	** Sequence**	**Tm**
Inner forward	5̕ ATGCAAATATCTGTCTGAAACGTTC	54.3

## RESULTS

Among the 51 patients studied, 28 (54.9%) subjects were male, and 23 (45.1%) subjects were female. Patients were aged between 15 and 55 years. Among the total patients, 22 (43.1%) subjects were splenectomized. Mean (SD) Hb levels in male and female patients were 9.21 1.48 g/dl and 8.77 1.49 g/dl, respectively. In addition, mean (SD) HbF levels in male patients were 83.37 28.74 g/dl and in female patients were 81.85 33.76 g/dl. Chi-square test showed that these differences between male and female were not statistically significant (*P* = 0.46 and *P* = 0.41, respectively).

**Fig. 2:**
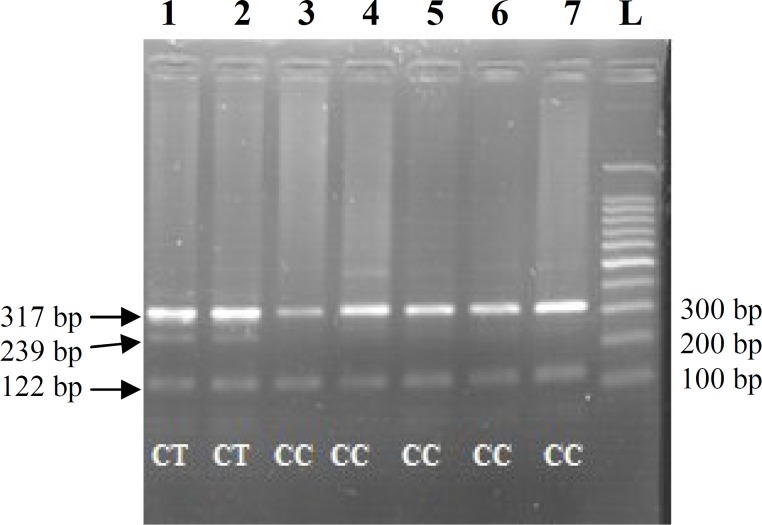
^Xmn1γG^ ^polymorphism genotyping^ ^using^ ^Tetra-Primer ARMS-PCR technique^ ^in seven patients with ^ ^-thalassemia intermediate. ^^Genotypes are indicated ^^below each lane. ^^XmnI^^ is a single nucleotide polymorphism (rs7482144) at the -158 site upstream region of the γ-globin gene^

The *Xmn1γ*^G^ polymorphism genotype was determined in patients using the Tetra-Primer ARMS-PCR technique (Fig. 2). Patients 1 and 2 had three bands, indicating the heterozygous genotype (C/T). Patients 3-7 had 122-bp and 317-bp bands; therefore, its genotype was homozygous (CC).

Among the 51 patients, 35 (68.6%) subjects were heterozygous (CT) and 16 (31.4%) subjects were homozygous (CC). The frequency of allele T in males and females was 34.8% and 33.9%, respectively. Chi-square test indicated that there was no significant correlation between the presence of allele T at the *Xmn1* polymorphic site and sex (*P* = 0.89).

As shown in Table 2, the Hb and HbF levels in the patients with heterozygous (CT) genotype were significantly higher (9.58 1.25 gm/dl and 89.30 21.87, respectively) than the homozygous (CC) genotype (7.94 1.34 gm/dl and 70.32 40.56, respectively). Based on independent *t*-test, the differences were statistically significant (*P* = 0.001 and *P* = 0.04, Table 2).

The prevalence of splenectomy in heterozygous (CT) genotype of *Xmn1* polymorphism was 15 (42.9%) and in homozygous (CC) state was 7 (43.8%). Chi-square test indicated no significant association between the presence of T allele at *Xmn1* polymorphic site and splenectomy (P = 0.59, Table 2).

Among the 51 patients studied, 30 (58.8%) subjects were under treatment with hydroxyurea (17 [56.7%] males and 13 [43.3%] females). Mean Hb levels before and after treatment were 8.47 1.36 gm/dl and 9.01 1.52 gm/dl, respectively. Using the paired sample *t*-test, the observed differences were statistically significant (*P* = 0.03, Table 3). Mean HbF levels before and after treatment were 81.87 26.95 and 86.11 28.00, respectively. Paired sample *t*-test showed that the differences were not statistically significant (*P* = 0.32, Table 3). 

**Table 2:** Parameters associated with *Xmn1* polymorphism in 51 patients according to independent t-test and Chi-square test

**Parameter**	***Xmn1*** **(CT)**	***Xmn1*** **(CC)**	***P*** ** value**
Hb (gm/dl)	9.58 ± 1.25	7.94 ± 1.34	0.001
HbF	89.30 ± 21.87	70.32 ± 40.56	0.04
Splenectomy (%)	15 (42.9)	7 (43.8)	0.59

Among the patients under treatment by hydroxyurea, 20 (66.7%) subjects were heterozygous (CT) and 10 (33.3%) subjects were homozygous (CC). In this study, we observed that Hb and HbF levels in the patients with heterozygous (CT) genotype (0.7 1.26 and 5.95 14.8, respectively) were increased more than the patients with homozygous (CC) genotype (0.26 1.43 and 0.8 1.31, respectively). Paired sample *t*-test showed that more elevation in total Hb level was statistically significant (*P* = 0.03, Table 4), but differences in HbF level were not statistically significant (*P* = 0.37, Table 4).

## DISCUSSION

β-thalassemia is the most common monogenic disease in humans. Genetic and non-genetic factors such as (CT) polymorphism administration of hydroxyurea have been reported to influence *γ-globin *gene expression and the severity of clinical symptoms of β-thalassemia [10]. *Xmn1γ*^G^ affects Hb and HbF levels only in erythropoietic stress conditions [11]. Some studies have reported that there is no association between the presences of T allele at this site and the reduction of the clinical symptoms in β-thalassemia intermediate patients 12, 13]. In the present study, association between *Xmn1γ*^G^ polymorphism and Hb/HbF levels and the effects of hydroxyurea on β-thalassemia intermediate patients in Isfahani population were studied by the Tetra-Primer ARMS-PCR technique.

The frequency of T allele at the *Xmn1* polymorphic site has been reported differently in various popularions, varying from 10-76.9% [13-22]. However, in the present study, the frequency of T allele at *Xmn1* polymorphic site in 51 patients with β-thalassemia intermediate was found 34%. 

Different studies have proved that the existence of T allele at *Xmn1* polymorphic site is associated with an increased amount of total Hb and HbF in intermediate β-thalassemia patients [22-25]. The presence of T allele in *Xmn1* polymorphic site reduces the binding of transcription silencers to the *γ-globin* gene promoter. Therefore, the *γ-globin* gene is reactivated in adult life in erythropoietic stress conditions [2, 7]. Numerous studies have revealed that there is a significant correlation between the occurance of T allele at *Xmn1* polymorphic site and increased amount of HbF and even reduction of severity of clinical symptoms in patients [6, 16, 20, 23-29]. However, some other studies have indicated that there is no association between the presence of T allele at this site and increased HbF level [15]. It has been also reported that there is no association between the presence of T allele at this site and the reduction of clinical symptoms in β-thalassemia intermediate patients [12]. In line with the majority of the first group, we have found that the levels of Hb and HbF are significantly increased in the presence of T allele at the *Xmn1* polymorphic site. These different results in various studies could be caused by the complexity of gene regulation pathways for *γ*-globin gene expression and also HbF levels [12]. Hydroxyurea is a chemical agent that may increase Hb and HbF levels. This effect can be exerted through* -globin* expression, and it seems to be associated with allele at the *Xmn1* polymorphic site [10, 17, 26, 30-32].

**Table 3:** Mean Hb and HbF levels in 30 patients under treatment with hydroxyurea according to paired sample *t*-test

**Mean level**	**Before** ** treatment**		**After treatment**		***P*** ** value**
Hb* (g/dl)	n = 20 (CT)	n = 10 (CC)	n = 20 (CT)	n = 10 (CC)	0.03
	8.47 ± 1.36		9.01 ± 1.52		
HbF**	n = 15 (CT)	n = 7 (CC)	n = 10 (CT)	n = 6 (CC)	0.32
	81.87 ± 26.95		86.11 ± 28.00		

* Of 30 patients, 20 and 10 patients were heterozygote (CT) and homozygote (CC), respectively;

** HbF information for 22 out of 30 patients (before receving hydroxyurea) and 16 out of 30 patients (after treatment with hydroxyurea) was prepared according to hospital files.

**Table 4:** Parameters associated with *Xmn1* polymorphism in 30 patients under treatment with hydroxyurea using paired sample *t*-test

**Parameter**	***Xmn1*** ** (CT)**	***Xmn1*** ** (CC)**	***P*** ** value**
Elevation of Hb levels with hydroxyurea	0.70 ± 1.26	0.26 ± 1.43	0.03
Elevation of HbF levels with hydroxyurea	5.95 ± 14.8	0.80 ± 1.31	0.37

In the present study, the frequency of *Xmn1* polymorphic site in 51 patients with β-thalassemia intermediate was determined, and its correlation with levels of Hb and HbF was analyzed. The results indicated that in the presence of T allele at *Xmn1* polymorphic site, the Hb and HbF levels were increased. In addition, the association between *Xmn1γ*^G^ polymorphism and the effect of hydroxyurea was studied. In the curren investigation, it has been demonstrated that in the patients carrying T allele, Hb and HbF levels are increased statistically, and they also response to hydroxyurea treatment better than patients without the T allele.
